# Efficacy of Biodentine as an Apical Plug in Nonvital Permanent Teeth with Open Apices: An In Vitro Study

**DOI:** 10.1155/2015/359275

**Published:** 2015-09-07

**Authors:** Mehmet Bani, Elif Sungurtekin-Ekçi, Mesut Enes Odabaş

**Affiliations:** ^1^Department of Pediatric Dentistry, Faculty of Dentistry, Gazi University, 06510 Ankara, Turkey; ^2^Department of Pediatric Dentistry, Faculty of Dentistry, Yeditepe University, 34728 Istanbul, Turkey

## Abstract

The aim of this study was to evaluate the apical microleakage of Biodentine and MTA orthograde apical plugs and to compare the effect of thickness of these biomaterials on their sealing ability. A total of eighty maxillary anterior teeth were used. The apices were removed by cutting with a diamond disc (Jota, Germany) 2 mm from the apical root end in an attempt to standardize the working length of all specimens to 15 ± 1 mm. Both materials were placed in 1–4 mm thickness as apical plugs root canal. Root canal leakage was evaluated by the fluid filtration technique. One-way ANOVA was used in order to determine normality of dispersal distribution of parameters; thereafter, results were analyzed by Kolmogorov-Smirnov test. Overall, between microleakage values of MTA and Biodentine regardless of apical plug thickness, no difference was observed. In terms of plug thickness, a statistically significant difference was observed between the subgroups of MTA and Biodentine (*p* < 0.05). The apical sealing ability of Biodentine was comparable to MTA at any apical plug thickness.

## 1. Introduction

Trauma or caries might result in pulp necrosis in young permanent teeth. Pulp necrosis in immature permanent teeth will eventually cause cessation of root closure and therefore root maturation. Thus, canal walls become thin and fragile and apex of tooth remains open. Under these circumstances, root canal instrumentation will not enable the achievement of an adequate apical stop [1–3].

Endodontic treatment of immature permanent teeth with open apices involves inducing of apical closure by apexification procedures [4]. Apexification with calcium hydroxide has been a proven approach and has been successfully performed over a long time. However, this method requires multiple treatment sessions and due to the presence of thin roots or prolonged exposure of root dentin to calcium hydroxide, the tooth will be more susceptible to root fracture [1, 5]. One visit apexification has an increasing popularity with the use of mineral trioxide aggregate (MTA) as an osteoconductive apical barrier [4, 6, 7]. The sealing ability and thickness of MTA apical plug have been mostly demonstrated as a successful performance [7, 8]. MTA is noncytotoxic and stimulates cementogenesis. Therefore, MTA is currently used as a root-end filling material in teeth with open apices [6–10].

On the other hand, MTA has been demonstrated to have some disadvantages including a long setting time, high cost, and potential for discoloration. It does not have good handling characteristics and its antibacterial properties are unpredictable [11–14]. To overcome these problems, a new biomaterial, Biodentine (Septodont, Saint Maur des Fosses, France), has been introduced. Biodentine is a new calcium silicate-based restorative cement with dentin-like mechanical properties. Biodentine consists of powder and liquid. The powder mainly contains tricalcium and dicalcium silicate (3CaO SiO_2_ and 2CaO SiO_2_), which is the main component of Portland cement, as well as calcium carbonate (CaCO_3_) and zirconium oxide. The liquid consists of calcium chloride (CaCl_2_·2H_2_O) solution with an admixture of polycarboxylate [13, 15–17]. Biodentine has a good sealing ability and has shown favorable biologic response as a pulp capping agent. It also has good mechanical properties; its setting time is 12 minutes and does not cause discoloration [17–19]. Biodentine has also endodontic indications similar to MTA and it can be used as a retrograde apical filling. However, there are few studies using Biodentıne as an apical barrier for the purpose of outcome comparison. And also no information exists regarding the effects of Biodentine as an apical barrier for one visit apexification treatment in teeth with open apices.

Therefore, the aim of this study was to evaluate the apical microleakage of Biodentine and MTA orthograde apical plugs and to compare the effect of thickness of these biomaterials on their sealing ability using the fluid filtration method.

## 2. Materials and Methods

A total of eighty maxillary anterior teeth were selected. Selected tooth samples all had single-rooted intact roots with completely formed apices. All teeth were examined in a stereomicroscope under ×16 magnification, and teeth with cracks, fractures, root canal calcification, and external root resorption were excluded. For disinfection, the specimens were stored in 5.25% sodium hypochlorite (NaOCl) for an hour and then placed in normal saline before the experiment.

The apices were removed by cutting with a water-cooling diamond disc (Jota, Germany) 2 mm from the apical root end in an attempt to standardize the working length of all specimens to 15 ± 1 mm. Subsequently, real root canal lengths were determined by manually inserting #15 K-files (Mani, Japan) into the canals, until the instrument tips were visible at the apical foramen. Apical instrumentation of roots was carried out with stainless steel files to a size of #40 K-file as a master apical file; subsequently, canals were flared up to #80 with the step back technique. A K-type patency file with a size of #15 was used for the canal preparation phase, and then canals were irrigated with 1.0 mL of 2.5% NaOCl. 17% ethylenediaminetetraacetic acid (EDTA) was used for 30 s after mechanical canal preparation, followed by 2.5% NaOCl for 30 s. A final flush with 5.0 mL of sterile saline solution (Farmence, Brazil) was performed. Samples were then randomly allocated to MTA group (*n* = 40) and Biodentine group (*n* = 40). The teeth were subdivided into 4 groups based on apical plug thickness (1, 2, 3, and 4 mm).

The roots were dried with #80 paper points (Ariadent, Iran) prior to treatment. MTA (ProRoot MTA, Dentsply, USA) and Biodentine (Septodont, Saint Maur des Fosses, France) were handled according to the manufacturer's instructions. The MTA and Biodentine were condensed up to the apical end with aid of plugger #3 and plugger #4 (Maillefer, Switzerland) with a rubber stop positioned at the root canal length and placed in 1–4 mm thickness as apical plugs root canal. Then, a moistened paper point was placed into the root canals and the density and thickness of apical plugs were confirmed by periapical radiograph. All canals were filled with a single tapered gutta-percha cone (Dentsply/Maillefer, Ballaigues, Switzerland) after an appropriate setting time suggested by the manufacturer. The sealer (Dentsply, Dentsply/Maillefer, Switzerland) was manipulated according to manufacturers' instructions and placed into the root canals with the aid of a #4 Lentulo spiral. Coronal access was sealed with composite resin (Clearfil Majesty, Dentsply, Switzerland).

All teeth were stored at 37°C in 100% humidity for 24 hours. After this period, the external root surfaces of the specimens were completely covered with three coats of nail polish, except for an area of 2.0 mm around the root apex.

The teeth were prepared for the evaluation of leakage 48 h later by the fluid filtration technique employing a pressure equivalent to 0.5 atmosphere, as described by Wu and Wesselink [20]. Four measurements were recorded for each tooth at 2-minute intervals over a period of 8 minutes. The amount of leakage was expressed as *μ*L·cmH_2_O^−1^·min^−1^. One-way ANOVA was used in order to determine normality of dispersal distribution of parameters; thereafter, results were analyzed by the Kolmogorov-Smirnov test. The critical value was set at 5% for all tests.

## 3. Results and Discussion

All specimens demonstrated variable amounts of apical leakage.  Table 1 shows mean microleakage values and standard deviations for MTA and Biodentine according to their plug thickness. Overall, a statistically significant difference was not observed between the microleakage values of MTA and Biodentine regardless of apical plug thickness (*p* > 0.05). The value of apical microleakage was significantly lower for 3 and 4 mm apical plugs than 1 and 2 mm subgroups of Biodentine and MTA (*p* < 0.05) (Table 1).

Various methods have been used to determine the sealing ability of apical barriers, such as bacteria and toxin infiltration method, fluid filtration method, dye penetration method, radioisotope, and the electrochemical method [21–23]. In the present study, fluid filtration method was used. Fluid filtration method carries many advantages compared with the other methods. With this method, samples are not destroyed, apical and coronal sealing are evaluated after a long period, the results are registered automatically avoiding operator errors, very little volumes can be recorded and repeated, measurements are more sensitive, and it is, therefore, widely accepted [20, 21, 23–25].

In the present study, the specimens were standardized regarding the root canal length and the diameter of apical opening. Standard preparation procedures and single tapered gutta-percha cone were also adopted in order to more closely simulate clinical procedures and to minimize operator variability.

Many studies have been published regarding the use of MTA in open apices for one visit apexification procedure [9–12, 14]. However, there is no information about the effect of the Biodentine on apical microleakage even if Biodentine has been used for other applications such as perforation repair and pulp capping [13, 26–29]. The present study aimed to compare the apical sealing abilities of Biodentine and MTA in teeth with simulated open apices.

The results of the present study led to a conclusion that both 1 and 2 mm apical plugs of Biodentine and MTA might be ineffective against apical leakage. However, 3 and 4 mm apical plugs of MTA gave satisfactory results, which is in agreement with previous studies [7, 21, 24, 30]. However, there are no studies for the purpose of outcome comparison wıth Biodentine. According to the results of the present study, the amount of apical microleakage was significantly lower for 3 and 4 mm apical plugs than 1 and 2 mm subgroups of Biodentine and MTA.

MTA is a type of hydraulic cement that can set in the presence of water. Hydraulic cements are finely ground materials that, when mixed with water, gradually set and harden in water. Biodentine was similar to MTA, with the exception of zirconium oxide added to the powder of Biodentine [16], as setting accelerator water reducing agent in liquid [26]. This reduces the setting time to 12 min and increases the compressive strength. Because of its high compressive strength, manufacturers recommend Biodentine to be used in endodontic treatments [16]. In the present study, Biodentine showed a success rate comparable to MTA when used for apical closure. Although MTA has good sealing properties when used as an apical plug, its handling properties and long setting time complicate its usage in orthograde endodontic therapy. When MTA is mixed with distilled water, it is difficult to handle and deliver the material through an orthograde direction [4]. On the other hand, Biodentine has easy handling characteristics and its placement is less time-consuming than MTA. Further clinical studies are recommended to better understand the performance of Biodentine used as an orthograde apical filling in teeth with open apices.

## 4. Conclusions

There were no study about the apical sealing properties of MTA and Biodentine for the purpose of outcome comparasion. According to the results of the present study, the apical sealing ability of Biodentine was similar to MTA at any apical plug thickness and it can be concluded that reduction of the apical plug thickness significantly increases apical microleakage. 3 and 4 mm thickness of apical plugs revealed a good sealing ability regardless of the tested biomaterials. The results of this study can help dentists for deciding apical plug thickness in open apices teeth.

## Figures and Tables

**Table 1:** Mean ± SD (×1000) apical leakage of MTA and Biodentine (*μ*L·cmH_2_O^−1^·min^−1^) (*n* = 10).

	1 mm	2 mm	3 mm	4 mm
MTA	2.39 ± 0.14^a^	1.98 ± 0.17^a^	0.67 ± 0.05^b^	0.56 ± 0.05^b^
Biodentine	2.03 ± 0.11^a^	1.85 ± 0.10^a^	0.71 ± 0.04^b^	0.60 ± 0.05^b^

The superscript letters (a and b) indicate significant differences between the tested thicknesses of biomaterials by one-way ANOVA and post hoc Kolmogorov-Smirnov test (*p* < 0.05).
